# Competition on presynaptic resources enhances the discrimination of interfering memories

**DOI:** 10.1093/pnasnexus/pgad161

**Published:** 2023-05-15

**Authors:** Chi Chung Alan Fung, Tomoki Fukai

**Affiliations:** Department of Neuroscience, City University of Hong Kong, Tat Chee Avenue, Kowloon Tong, Hong Kong, China; Neural Coding and Brain Computing Unit, Okinawa Institute of Science and Technology Graduate University, 1919-1 Tancha, Onna-son, 904-0495 Okinawa, Japan; Neural Coding and Brain Computing Unit, Okinawa Institute of Science and Technology Graduate University, 1919-1 Tancha, Onna-son, 904-0495 Okinawa, Japan

**Keywords:** adult neurogenesis, synaptic competition, pattern separation, computational model, Hebbian-like plasticity

## Abstract

Evidence suggests that hippocampal adult neurogenesis is critical for discriminating considerably interfering memories. During adult neurogenesis, synaptic competition modifies the weights of synaptic connections nonlocally across neurons, thus providing a different form of unsupervised learning from Hebb’s local plasticity rule. However, how synaptic competition achieves separating similar memories largely remains unknown. Here, we aim to link synaptic competition with such pattern separation. In synaptic competition, adult-born neurons are integrated into the existing neuronal pool by competing with mature neurons for synaptic connections from the entorhinal cortex. We show that synaptic competition and neuronal maturation play distinct roles in separating interfering memory patterns. Furthermore, we demonstrate that a feedforward neural network trained by a competition-based learning rule can outperform a multilayer perceptron trained by the backpropagation algorithm when only a small number of samples are available. Our results unveil the functional implications and potential applications of synaptic competition in neural computation.

**Significance Statement**

Adult neurogenesis in the dentate gyrus (DG) is critical for separating interfering memories. At the same time, adult-born DG neurons compete with existing DG neurons for input connections from the entorhinal cortex. However, the contribution of synaptic input competition to information processing is still largely omitted. This work offers a simple and effective toy model to show the potential of synaptic competition for neuronal inputs. In addition to the implication in computational Neuroscience, we have shown that the neural network trained by long-term potentiation with synaptic competition has a better performance than a multilayer perceptron trained by backpropagation in a similar network setting. This result highlighted the contribution of synaptic competition in information processing.

## Introduction

Long-term synaptic plasticity such as spike-timing-dependent plasticity (STDP) (1–3) has been considered to play a vital role in memory formation (4). STDP suggests how a neuronal network could learn the appropriate connection strengths from temporal correlations between presynaptic and postsynaptic neuron pairs. A simplified picture proposed by Hebb suggests that neurons with positively correlated activities should be connected more strongly and vice versa (5). This rule is known to be the Hebbian learning rule. Although the Hebbian rule is a good summary of long-term synaptic plasticity, the entire picture of the learning process in neuronal networks has yet to be completed (6).

Neurons competing with other neurons for input connections is another form of synaptic updating rules that may cause long-term changes in neuronal connections. “Synaptic competition” in this work refers to this kind of competition for presynaptic input. Synaptic competition likely plays a crucial role in neural information processing in the brain. For instance, ganglion cells compete for synaptic input during the development of neural circuits (7). Moreover, recent evidence suggests that synaptic competition occurs during adult neurogenesis in the hippocampus (8, 9). The ablation of adult neurogenesis may impair some cognitive functions and cause mental disorders (10), suggesting its importance in supporting normal brain functions.

The dentate gyrus (DG) (11–14), an entry terminal of the hippocampal formation, and the olfactory bulb (15, 16) are also known to undergo adult neurogenesis. DG receives excitatory synaptic input from the entorhinal cortex (17) and projects excitatory output to the hippocampal area CA3 (18), and hence is a vital relay in the trisynaptic circuit (19). Various factors regulate the birth rate of new neurons (11–14), making the functional roles of adult neurogenesis complex. Generally, however, adult neurogenesis is essential for behavioral tasks that require the discrimination of similar memories (9). Mice with ablated adult hippocampal neurogenesis fail to discriminate memories with significant mutual interference (20–23). Similar negative effects of the ablation or the suppression of adult neurogenesis were observed in other cognitive tasks (21, 24–27). Furthermore, adult neurogenesis may reduce anxiety-like behaviors (10) as pharmacologically diverse antidepressants promote adult neurogenesis in rodents (28–31), nonhuman primates (32), human postmortem brain tissue (33, 34), and human hippocampal progenitor cells in vitro (35).

Adult neurogenesis has not been a topic of extensive computational studies, but several computational models already exist in the literature. The previous models focused on the roles of neuronal turnover and synaptic plasticity in memory processing without clarifying the essential role of synaptic competition. A network model was proposed to study the effects of neuronal turnover and Hebbian learning on discriminating similar memory patterns (36). However, the model did not address the computational implications of synaptic competition. Another modeling study considered most biological features of adult neurogenesis, although the computational advantage of synaptic competition was not explicitly examined (37). Yet another model suggested, without taking synaptic competition into account, that neuronal turnover significantly reduces the dimension of memory representations in the neural network of DG (38).

This study aims to clarify whether and how synaptic competition improves pattern separation. We begin by comparing performance in pattern separation between various learning rules, including synaptic competition and the conventional weight-updating rules of long-term potentiation (LTP) and depression (LTD). We first consider toy problems with interfering memory patterns to show the different properties of different learning rules, and propose an unsupervised learning algorithm that implements synaptic competition. Then, we train a multilayer network adopting this learning rule on a real-world task, pattern separation on the MNIST data set. We demonstrate that our model can outperform a multilayer perceptron trained by the backpropagation algorithm, which is the state-of-art supervised learning algorithm for pattern recognition (39). Notably, the synaptic competition-based learning rule works exceptionally well when only a small number of samples is available for training. Thus, our results suggest that the virtue of synaptic competition during adult neurogenesis resides in memory formation with small sample sizes or small trial numbers—a brain feature superior to artificial intelligence models.

## Results

### LTP alone overgeneralizes overlapping patterns

**Fig. 1.**
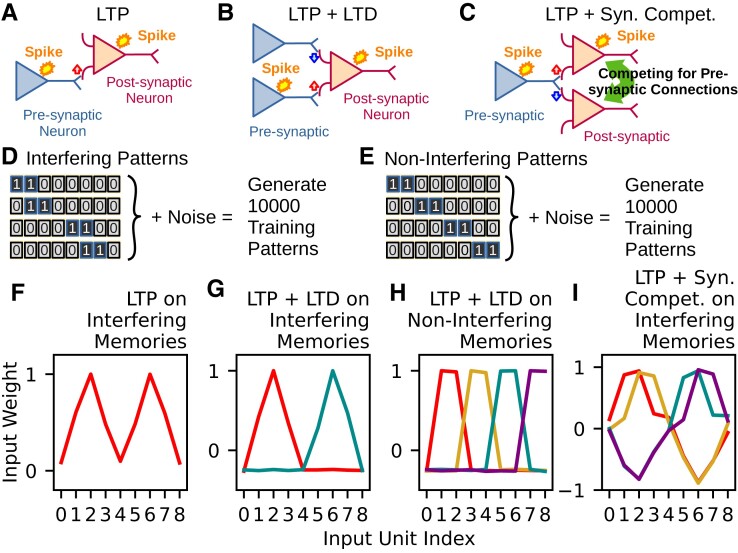
Synaptic plasticity and synaptic competition, and the trained input weights. A). LTP is illustrated schematically. B) Combinations of LTP and LTD occur on a single neuron. C) LTP is combined with synaptic competition in which synapses compete for presynaptic resources. D) Fundamental patterns to generate training patterns for the investigation of interfering memories. E) Fundamental patterns to generate training patterns for the investigation of noninterfering memories. F) A representative profile trained by LTP with samples derived from the overlapping fundamental patterns given in Eq. 1. G) Representative profiles trained by LTP and LTD with overlapping fundamental patterns. Two weight profiles respectively peaking at input units 2 (red) and 6 (cyan) are obtained. H) Representative profiles trained by LTP and LTD with samples of the nonoverlapping fundamental patterns given in Eq. 8. Four distinct profiles with different peak locations appear. I) Representative profiles trained by LTP and synaptic competition with samples of the overlapping fundamental patterns. The profiles are categorized into two groups (red (peaked at input unit 2 and left-skewed) and orange (peaked at input unit 2 and right-skewed) vs. cyan (peaked at input unit 6 and left-skewed) and purple (peaked at input unit 6 and right-skewed)) according to the grouping of excited and inhibited input units.

To begin with, we study a toy model to clarify how LTP modifies synaptic weights for overlapped patterns. As illustrated in Fig. 1A, we model the LTP by the Hebbian learning rule: when presynaptic and postsynaptic neurons are coactivated, synapses between them should be strengthened. We will describe the mathematical details of the learning rule later. Each sample pattern ξ for training is generated as the sum of a pattern selected randomly from four fundamental patterns (Fig. 1D) and a noise vector with a magnitude *η* (see Methods in Supplementary Material). These fundamental patterns are mathematically given as


(1) $${\bar{\xi }}^{\left(p\right)}=\left\{ \begin{array}{cc} (0,1,1,0,0,0,0,0,0{)}^{T} & \text{if }p=0\\ (0,0,1,1,0,0,0,0,0{)}^{T} & \text{if }p=1\\ (0,0,0,0,0,1,1,0,0{)}^{T} & \text{if }p=2\\ (0,0,0,0,0,0,1,1,0{)}^{T} & \text{if }p=3 \end{array}\right..$$

Note that patterns generated from pattern p=0 overlap with those from pattern p=1 (group 1). So are pattern p=2 and pattern p=3 (group 2). There is no overlaps between patterns belonging to the different groups.

To implement the Hebbian learning model, we consider a two-layer neural network in which the first layer is an input terminal and the second layer is an output terminal (see Methods in Supplementary Material). The activity of the *j*th input unit is denoted by $x_{\;j}$, while that of the *i*th stochastic output unit by $y_{i}$ (Methods), and the weight of synapse connecting the output unit *i* and input unit *j* is denoted by $w_{ij}$. During learning, the synaptic weights evolve as


(2) $$w_{ij}(t+1)=w_{ij}(t)+\gamma y_{i}(t)x_{\;j}(t),$$

where *t* specifies the time of presentation of the *t*th sample pattern and γ=0.1 is the learning rate. The values of $w_{ij}$ are initially zero. In the training process, sample patterns are presented in a random sequence such that no class labels can be inferred in the sequence. In each iteration, we normalize input synaptic weights on each output unit if the maximum weight exceeds 1 on the output unit:


(3) $$\begin{array}{rl} w_{ij}(t+1) & \to \frac{w_{ij}(t+1)}{\max_{j}w_{ij}(t+1)},\\ & \;\text{if }\max_{\;j\in \mathrm{input}\;\;\;\mathrm{units}}w_{ij}(t+1)>1. \end{array}$$

In this setting, we can easily deduce the trained weights; that is, changes in input synaptic weights averaged over time should be proportional to the averaged input patterns:


(4) $$\langle (w_{i}(t+1)-w_{i}(t))\rangle ∼\langle x\rangle ,$$

for an arbitrary output unit *i*. This implies that the presentation of an input pattern rotates the synaptic weight vector in the direction of the input pattern. Thus, for the patterns given in Eq. 1, the resultant weights will be given as


(5) $${\begin{array}{c} w \end{array}}_{i}(t)\to \left(0,\frac{1}{2},1,\frac{1}{2},0,\frac{1}{2},1,\frac{1}{2},0\right).$$

As the number of trained samples increases, input weights onto output units will become similar to each other. The equilibrium weights will show no difference between the two fundamental patterns in Eq. 1. Fig. 1F shows typical examples of input synaptic weights obtained for a representative neuron by numerical simulations. The numerical results agree well with those predicted in Eq. 5. The mean and standard deviation across neurons (Fig. S1A and B, Supplementary Material) suggested that input synaptic weights on other neurons are almost identical to those shown in Fig. 1F. The cosine similarities between input weights and fundamental patterns shown in Fig. S2A of Supplementary Material confirmed that these input weights have no preferences for any of the training patterns. Therefore the neuronal activities corresponding to the fundamental patterns p=1,2,3 and 4 are almost the same. By definition, the entropy of those neuronal activities is zero. Hence, the mutual information between the output y and input x will also vanish.

### LTP and LTD jointly separate nonoverlapping patterns

The LTP-like Hebbian learning rule tends to overgeneralize the training patterns. The output neurons with input synaptic weights trained by LTP alone (Fig. 1F) could not distinguish different fundamental patterns. Here, we incorporate LTD to examine if combining LTP and LTD in learning rules improves the resolution of separation between patterns. Fig. 1B schematically illustrates the LTD used in this study.

To this end, we modify the learning rule in Eq. 2 as follows:


(6) $$w_{ij}(t+1)=w_{ij}(t)+\gamma y_{i}(t)[x_{\;j}(t)-\theta ],$$

where the additional term θ controls the intensity of LTD. Input synaptic weights undergo LTD when the corresponding presynaptic activities are weaker than the threshold value. Eq. 6 has a similar structure to Eq. 2, and the average change of input weights would be


(7) $$\langle (w_{i}(t+1)-w_{i}(t))\rangle ∼\langle x-\theta \rangle .$$

The above equation is similar to Eq. 4 except that inhibitory connections can emerge due to the *θ* term. Now, input patterns corresponding to p=0,1 and those corresponding to p=2,3 excite nonoverlapping input-unit subgroups. Therefore, Eq. 7 implies that input weights and output neurons will evolve into two subgroups. The simulation results shown in Fig. 1G agree with this prediction. The cosine similarities between input weights and the four fundamental patterns shown in Fig. S2B and C of Supplementary Material indicate that input synaptic weights selectively tune to either group of patterns, p=0,1 or p=2,3.

The results suggest that LTD helps output neurons to distinguish the distinct groups of input patterns (i.e. the group for p=0,1 and that for p=2,3). From another perspective, we may say that LTD generalizes patterns with a substantial overlap (i.e. patterns for p=0 and those for p=1, etc.) into the same category. The means and standard deviations of input weights of the different groups are shown in Fig. S1C and D of Supplementary Material, respectively. Input weights take identical values on neurons tuned to the same group of overlapping patterns.

The above results showed that LTD can separate nonoverlapping groups of patterns. To further verify the implications of LTD in pattern separation, we trained the toy model with the fundamental patterns that are not mutually overlapped (Fig. 1E):


(8) $${\bar{\xi }}^{\left(p\right)}=\left\{ \begin{array}{cc} (0,1,1,0,0,0,0,0,0{)}^{T} & \text{if }p=0\\ (0,0,0,1,1,0,0,0,0{)}^{T} & \text{if }p=1\\ (0,0,0,0,0,1,1,0,0{)}^{T} & \text{if }p=2\\ (0,0,0,0,0,0,0,1,1{)}^{T} & \text{if }p=3 \end{array}\right..$$

Input weights are trained by Eq. 6. Since the sample patterns corresponding to p=0,1,2 and 3 have no overlaps and they are presented in a random sequence, output nodes inhibited by a sample pattern can be excited by some other patterns. As a result, input weights separate into four groups:


(9) $$w_{i}∼\left\{ \begin{array}{c} (-\theta ,1-\theta ,1-\theta ,-\theta ,-\theta ,-\theta ,-\theta ,-\theta ,-\theta {)}^{T}\\ (-\theta ,-\theta ,-\theta ,1-\theta ,1-\theta ,-\theta ,-\theta ,-\theta ,-\theta {)}^{T}\\ (-\theta ,-\theta ,-\theta ,-\theta ,-\theta ,1-\theta ,1-\theta ,-\theta ,-\theta {)}^{T}\\ (-\theta ,-\theta ,-\theta ,-\theta ,-\theta ,-\theta ,-\theta ,1-\theta ,1-\theta {)}^{T} \end{array}\right.$$

Input weights resultant from the simulation are shown in Fig. 1H, and Fig. S1E and F of Supplementary Material. As suggested by Eq. 9, the numerical results yielded four types of input weights. Fig. S2D to G demonstrate that each type of input weights has a significant overlap with a different fundamental pattern. These analytical and theoretical results revealed that the combination of LTP and LTD can organize input weights to distinguish nonoverlapping patterns. This result also suggests that the learning rule defined in Eq. 6 does not limit the number of classes the network can separate. However, as shown previously, this learning rule generalizes but cannot distinguish overlapping patterns.

### Synaptic competition differentiates overlapping patterns

Learning rules combining LTP and LTD tend to overgeneralize similar input patterns and cannot differentiate overlapping input patterns, e.g. Eq. 1. As input patterns generally have overlaps in many real-world problems, this result severely limits the validity of the feedforward network model in pattern separation. Another type of weight updating rule is necessary to differentiate mutually interfering patterns.

The extreme overgeneralization may occur when different input patterns do not compete with each other for getting resources for learning, that is, synaptic connections. Synaptic learning rules without competition are also far from realistic as they assume that infinite resources are available at each synapse. In reality, there should be an upper bound of the number and/or total strength of synaptic connections.

We searched for an efficient mechanism of synaptic competition by modeling some properties of adult neurogenesis, which occurs in the DG and olfactory bulb. Adult neurogenesis is considered crucial for pattern separation in episodic memory and odor discrimination. In those brain regions, the maturation of newly born neurons is an important process for developing synaptic connections from neurons in input layers. During this process, newly born neurons compete for input synaptic connections with existing neurons. Furthermore, the expansion of synaptic contacts terminates on matured newborn neurons. Below, we implement these two properties in the toy model.

Considering that the shortage of resources would affect the growth of synapses, we may model the maturation of synaptic contacts by terminating LTP if the sum of input weights on each neuron exceeds a certain threshold. We can describe the maturation process by the following equation:


(10) $$\Delta {\tilde{w}}_{ij}(t)=\left\{ \begin{array}{ll} y_{i}(t)x_{\;j}(t), & \text{if }\sum_{j\in \mathrm{input}\;\;\;\mathrm{units}}w_{ij}<\Theta \\ 0 & \text{otherwise}. \end{array}\right.$$

This equation describes the degradation of LTP after the maturation of DG neurons: as in mature granule neurons, only bursts of postsynaptic action potentials can induce LTP (40).

We model synaptic competition for presynaptic resources as


(11) $$\Delta w_{ij}(t)=\Delta {\tilde{w}}_{ij}(t)-\langle \Delta {\tilde{w}}_{ij}(t)\rangle_{i},$$

where $\langle \cdots \rangle_{i}$ implies averaging across the output neurons projected to by input neuron *j*. Here, This equation should not be confused with homeostatic plasticity regulated by the postsynaptic activity (41). The updating rule for $w_{ij}$ is given as


(12) $$w_{ij}(t+1)=w_{ij}(t)+\gamma \Delta w_{ij}(t).$$

Then, we renormalize all synaptic weights from input unit *j* if the largest weight from the unit exceeds unity:


(13) $$\begin{array}{rl} w_{ij}(t+1) & \to \frac{w_{ij}(t+1)}{\max_{i\in \mathrm{output}\;\;\;\mathrm{units}}w_{ij}(t+1)}\\ & \;\mathrm{if}\;\max_{i\in \mathrm{output}\;\;\;\mathrm{units}}w_{ij}(t+1)>1. \end{array}$$

Unlike Eq. 3, Eq. 13 normalizes the net output of input units.

While Eq. 10 integrates all input weights that can excite an input unit, Eq. 11 induces competition across postsynaptic neurons for input weights from the same input neurons. The competition breaks the symmetry of input weights in Fig. 1F during training. Fig. 1I reveals four representative tuning curves. Two of them have peaks at the input unit 2 and another pair at the input unit 6. This configuration, with peaks at units 2 and 6, is similar to that in Fig. 1G. However, the competition between input weights segregates input units, inducing a broader tuning pattern in the output neuron population. Consequently, we can now categorize input weights in Fig. 1I by their locations of the second-highest values. The means and standard deviations of the input weights for different categories are shown in Fig. S1E and F of Supplementary Material, respectively. In addition, the cosine similarities between the representative input weights and fundamental patterns shown in Fig. S2H to K of Supplementary Material suggested that input weights obtained by LTP and synaptic competition have different preferred fundamental patterns, even when some of the patterns are overlapped. These results have important implications for pattern separation as they suggest synaptic competition, unlike LTD, enables the network model to differentiate similar input patterns.

### Principal component analysis for synaptic competition

**Fig. 2.**
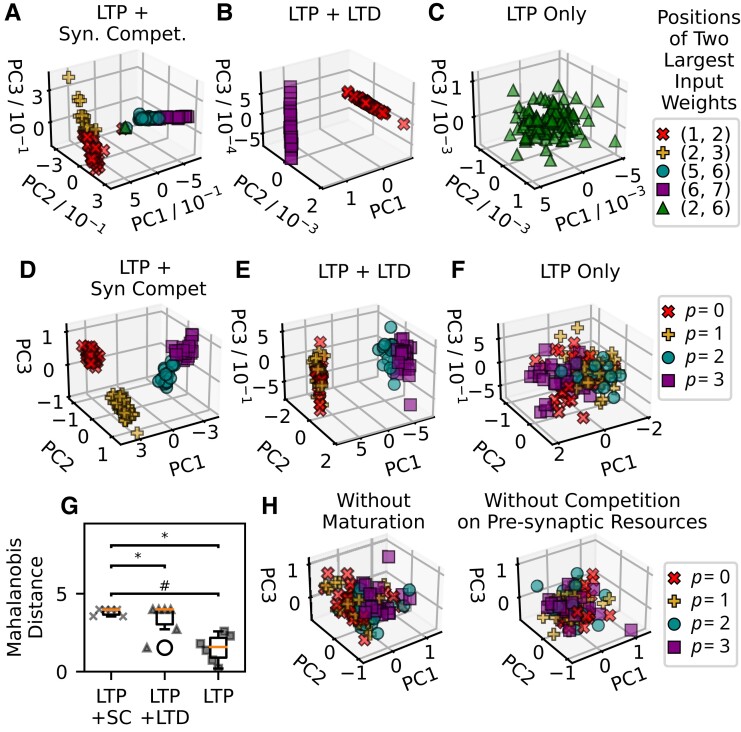
Principal component analysis (PCA) of input synaptic weights and activities of output units after training on interfering patterns. A) The input weights trained by LTP and synaptic competition are projected on the first three leading principal components (PCs), i.e. PC1, PC2, and PC3. B) Input weights trained by LTP and LTD are plotted in the space spanned by the leading PCs. C) Input weights trained by LTP only are plotted in the space spanned by the leading PCs. D) The outputs of neurons trained by LTP and synaptic competition encoding different preferred fundamental patterns are plotted in the 3D space of the leading PCs. E) A similar plot to (C) for a network trained by a combination of LTP and LTD. F) A similar plot to (C) for a network trained by LTP only. D–F) Pseudocolor code indicates the preferred fundamental patterns of output neurons. G) Box plots of Mahalanobis distances between neuronal activities corresponding to fundamental patterns for different learning rules: LTP and Synaptic Competition (LTP + SC), LTP and LTD (LTP + LTD), and LTP only (LTP). Crosses, triangles, and boxes represent the Mahalanobis distances between the neuronal activity clusters corresponding to six pattern pairs: From left to right, they are (0,1), (0,2), (0,3), (1,2), (1,3) and (2,3). #: Wilcoxon Signed-Ranks Test, P<0.05. *: *F*-test of equality of variances, P<0.01. H) Left: Projections of neuronal activities on the PC space in a toy model trained by synaptic competition without the maturation term. Right: Neuronal activities are projected on the PC space in a toy model trained by synaptic competition without competition for presynaptic resources.

Here, we used the standard principal component analysis (PCA) without whitening and show the resultant principal components (PCs) corresponding to the three largest eigenvalues. Fig. 2A shows the projections of input weights on the first three principal components in a 3D plot.

In Fig. 2A, we labeled the projected input weights trained by LTP and synaptic competition according to the locations of the two largest synaptic weights. Along the first principal component, the input weights peaked around input unit 2 and those peaked around input unit 6 are clearly separated (Fig. 1I). Then, the second and third principal components, (PC2) and (PC3), further separate input weights peaked around input unit 2, particularly those having the two largest weights at input units (1,2) and (2,3). The same subspace also segregates input weights having the two largest weights at (5,6) and (6,7).

Training by LTP and LTD without synaptic competition segregates the input weight into four groups (Fig. S3A, Supplementary Material) in the case of noninterfering fundamental patterns (Eq. 8). However, similar training on interfering patterns can only segregate the input weights into two groups (Fig. 2B). In this case, the input weights have the two largest weights at input units (1,2) or (6,7) (Fig. 1G). The differences between overlapping patterns (p=0/1 and p=2/3) are largely ignored. Moreover, training by “LTP only” generates a single cluster of the input weights in the space spanned by their principal components (Fig. 2C), agreeing with the results in Fig. 1F, and Fig. S1A and B of Supplementary Material. Altogether, our results clarify the crucial role of synaptic competition in separating similar patterns.

To further confirm the unique role of synaptic competition in pattern separation, we conducted PCA on output neuronal activities, $y_{i}$, obtained for patterns generated from the fundamental patterns. When the network model is trained by LTP and synaptic competition, the projected neuronal activities corresponding to different (both overlapping and nonoverlapping patterns) fundamental patterns are clearly distinguished (Fig. 2D). However, if the network model is trained by LTP and LTD, they can only separate nonoverlapping patterns (Fig. 2E). Output activities are significantly overlapped for overlapping fundamental patterns. If the fundamental patterns are not mutually overlapped (i.e. Eq. 8), there is a clear separation between neuronal activities corresponding to different fundamental patterns (Fig. S3B, Supplementary Material). This result suggested that the inability of LTP and LTD to distinguish overlapped patterns is not due to the capacity of the learning rule and the network.

In a network trained only with LTP, projected neuronal activities generally fall into a single cluster, implying that such a network has the little capability of pattern separation (Fig. 2F). To make a quantitative comparison between the three learning rules, we show Mahalanobis distances between the neuronal activities corresponding to different fundamental patterns in Fig. 2G. These results indicate that training with LTP and LTD can separate neuronal activities for nonoverlapping patterns, while training with “LTP only” shows much poorer performance in pattern separation. In contrast, training with LTP and synaptic competition can separate neuronal activities for different fundamental patterns regardless of whether they are overlapped or nonoverlapped. These results further confirmed that synaptic competition greatly enhances pattern separation in the toy model.

### Distinct roles of maturation and competition in pattern separation

The synaptic competition rule proposed above consists of two components: maturation, i.e. Eq. 10, and competition for presynaptic resources, i.e. Eq. 11. Both maturation and competition on presynaptic resources are indispensable for pattern separation shown in Fig. 2D. To see this, we modify the learning rule defined by Eqs. 0–2. To examine the importance of maturation, we replace Eq. 10 with the simplest Hebbian rule defined in Eq. 2. Similarly, to examine the importance of competition, we drop the second term in the right-hand side of Eq. 11. As shown in Fig. 2H, neither of the modified models can perform pattern separation at a satisfactory level: neuronal activities projected on the leading principal components exhibit a single cluster in each scenario.

We further analyze the different roles of maturation and synaptic competition. In the absence of synaptic competition, changes in synaptic weights are described by Eq. 4 and hence the resultant input weights are incapable of separating input patterns. On the other hand, if maturation is absent, by letting $w_{ij}>0$ and assuming a rectified linear activation function, we can derive the following energy function of $w_{ij}$s on output neuron *i*:


(14) $$\begin{array}{rl} E_{i} & =-\frac{1}{2}\sum_{\;j}[w_{ij}-\langle w_{kj}\rangle_{k\neq i}{]}^{2}\langle x_{\;j}^{2}\rangle \\ & \;-\sum_{l<j}[w_{il}-\langle w_{kl}\rangle_{k\neq i}][w_{ij}-\langle w_{kj}\rangle_{k\neq i}]\langle x_{l}x_{\;j}\rangle , \end{array}$$

where $\left\langle w_{kj}\rangle_{k\neq i}\equiv \sum_{k\neq i}w_{kj}/(N-1\right)$ is the mean weights from input neuron *j* to output neurons other than neuron *i*. The neuron-specific energy function indicates that input weights projecting to the same output neuron are positively coupled. If input weights grow dominantly on output neuron *i*, synaptic competition will strongly suppress other weights as $\langle w_{kj}\rangle_{k\neq i}$ can become arbitrarily large. Therefore, synaptic competition allows only the earliest-growing neurons to get all presynaptic resources. To show this, we plotted the energy function for the simplest case of two output neurons (Fig. S4, Supplementary Material). When the average weight on output neuron 1 is small, i.e. $\langle w_{1j}\rangle_{j}\approx 0$, input weights can grow on output neuron 2 without limitation, further suppressing the weight growth on neuron 1. If only a few output neurons dominate input weights, output neurons cannot differentiate input patterns. The maturation rule ceils the unlimited growth of such synapses to increase the diversity of output neurons.

### Synaptic competition facilitates pattern separation with small sample sizes

The toy model demonstrated the unique role of synaptic competition in discriminating interfering memory patterns. However, the toy model trained to discriminate only four patterns is too simple to claim the validity of synaptic competition for pattern separation. One may ask whether the competition-based learning rule works similarly well on real-world classification tasks with more complex input patterns. In the following, we train a feedforward network model by synaptic competition to examine whether the network can perform the classification of hand-written digits.

**Fig. 3.**
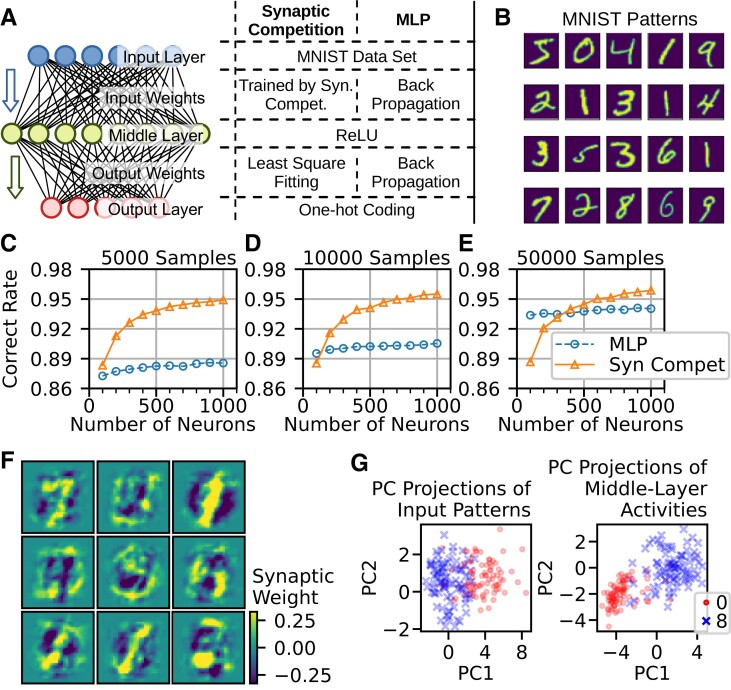
Performance test on a real-world classification task. A) The structures of three-layer neuronal networks are compared. A competition-based model was trained by synaptic competition and least-square fitting, while a multilayer perceptron (MLP) was trained by the backpropagation algorithm. B) Samples of hand-written digits in the MNIST data set. C–E) Correct rates of the competition-based model and MLP. The two networks were trained with 5,000 (C), 10,000 (D), and 50,000 (E) samples from the MNIST data set. F) Examples of trained input weights on the middle layer are shown for the competition-based network model in Fig. 3A. Twenty-five more examples are shown in Fig. S5 of Supplementary Material. G) Left: PCs of input patterns are shown for hand-written digits 0 (red dots) and 8 (blue crosses). Right: PCs of neuronal activities are shown for middle-layer neurons with a preference for hand-written digits 0 and 8.

To this end, we consider three-layer feedforward neuronal networks equipped with synaptic competition and the MNIST database of hand-written digits (from 0 to 9). We also consider a multilayer perceptron (MLP) for comparison in performance. The outline of our comparative study is explained in Fig. 3A. Both our feedforward neuronal networks for synaptic competition and MLP have $28^{2}$ units in their input layers and ten units in their output layer to discriminate the ten digits. Thus, the output layer supports one-hot coding, which gives a winner-take-all binary representation to each digit. In both models, neurons are rectified linear units (ReLU), that is, the activation function of neurons is a rectified linear function. The number of neuronal units in the middle layer is a parameter crucial for the comparison between the models. The differences between the two networks are the learning rules to train input and output weights. The weights are updated in the MLP network by the error-backpropagation algorithm (see Methods in Supplementary Material). In the network for synaptic competition, input weights were updated by synaptic competition, while the least-square fitting determined output weights after the training of input weights (see Methods in Supplementary Material).

We found that the synaptic competition-based learning rule boosts classification performance, especially when the sample size is small. There are 70,000 hand-written digits in the MNIST data set. Some examples are shown in Fig. 3B. In this work, we trained the network models on relatively small subsets of the data set with 5,000, 10,000, and 50,000 samples. Machine learning often requires big data, which severely limits the applicability of such algorithms. Therefore, we are particularly interested in the situation where only a small number of samples are available for learning. Furthermore, each input pattern is exposed only once to the network model with synaptic competition but five times in total to the MLP network. Under this condition, which is more stringent for the competition-based network model than for the MLP network, we trained both network models with different sizes of the middle layer. To our surprise, on the smallest sample data, the network with synaptic competition always outperforms the MLP network for all sizes of the middle layer (5,000 samples, Fig. 3C). The advantage of the competition-based network persists for larger sizes of samples (10,000 samples and 50,000 samples) with reasonably sufficient sizes of the middle layer (Fig. 3D and E). These results support the competency of competition-based learning rules in real-world classification tasks. To see if the synaptic competition rule can separate more classes, we trained the network using a data set combined with the MNIST data set and the Kuzushiji-MNIST data set (42). The combined data set contained a total of 140,000 samples from 20 classes. Fig. S6A displays examples of the training patterns. High classification performance by synaptic competition is largely preserved for the extended data set (Fig. S6B, Supplementary Material: c.f. Fig. 3C to E), suggesting the potential of the synaptic competition rule in categorizing many classes.

**Fig. 4.**
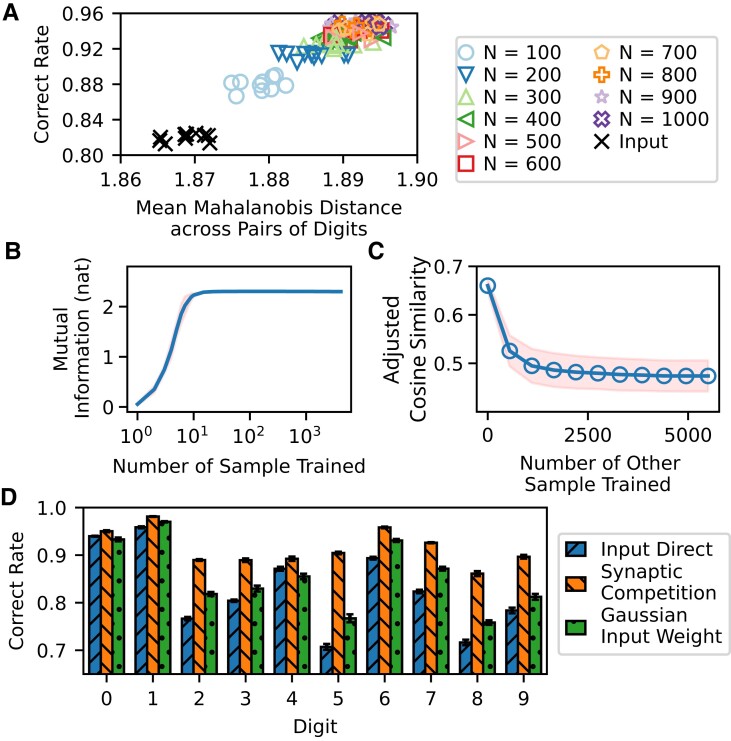
Analysis of representations of digits in the different layers of the competition-based network model. A) Correct rate versus separation of digit pairs in the competition-based network model. The relation between the correct rate and Mahalanobis distances between digit pairs for different *N*s are shown. In addition, the correct rate and Mahalanobis distance for input patterns are also plotted for comparison (black crosses). For each *N*, there are results from ten independent simulations. The number of samples was 5,000 for all simulations. B) Mutual information between activities of middle-layer neurons and input pattern labels. The entropy was defined by Shannon entropy and calculated by the histogram method. After training with a certain number of samples (horizontal axis), the mutual information was calculated using the whole data set. Line: Mean mutual information observed from simulations. Shaded area: the corresponding sample standard deviation. C) Adjusted cosine similarity between neuronal responses to a digit immediately after training on it and those to that digit after training on samples of other digits. The adjusted cosine similarity is defined by a normalized inner product of two vectors subtracted by a normalized inner product of element-shuffled versions of the two vectors. We used the adjusted cosine similarity to reduce artifacts arising in the overlaps from the nonnegativity of neural activity. Circles: Mean adjusted cosine similarity between neuronal activities observed from simulations. Shaded area: the corresponding sample standard deviation. D) Performance comparison between the different algorithms. Separate comparisons were made by using 5,000 samples of each digit class. The number of units in the middle layer was 200 for synaptic competition and input weights were Gaussian distributed. Error bars indicate the standard deviations of means over 10 independent trials.

We demonstrate how synaptic competition provides an improved cue for classifying hand-written digits of the MNIST data set. In the synaptic competition-based model, the tuning profiles of input weights on individual middle-layer neurons show interesting features. Fig. 3F shows examples of the tuning profiles, which look like “blurred” digits. Unlike other classification algorithms, the digits the neurons have learned are not obvious from the tuning profiles. The configuration of excitatory connections likely captures only a tiny portion of each digit, implying that the trained neurons are tuned to some strokes of the digits. We further conducted PCA of neuronal activities in the network trained by synaptic competition. Fig. 3G displays respectively the input pattern and middle-layer activity distributions for digits 0 and 8 projected on the two-dimensional space spanned by their first two PCs. While the distributions of the two digits are barely separated in the input layer, they are better separated in the middle layer. Besides the two lowest-order components, Fig. S7 of Supplementary Material suggests that some higher order components also contribute to pattern classification. Furthermore, Fig. 4A compares the correct rate and the average Mahalanobis distances between activities of digit pairs in input and middle layers for different *N*. This quantitative comparison strongly supports that synaptic competition expands separations between representations corresponding to different groups. Also, it confirms the link between pattern separation and classification performance.

We also investigated the time course of the learning process by measuring the mutual information between neuronal activities and class labels as a function of time (Fig. 4B). The result suggests that the synaptic competition rule enables the network to generate rich representations of input patterns. Actually, the mutual information rapidly reached the theoretical maximum value of ln10∼2.3 as the number of training samples increased. Thus, the training rule allows the network to collect the necessary information for classification in the early stage of learning.

We further tested how the learned patterns are degraded over time as novel patterns are encoded in the network. It is difficult to assess this property accurately in random sequential training on the MNIST data set. Therefore, we first trained the network on patterns of a particular digit and then trained the same network on those of other untrained digits. We measured the degree of degradation with the adjusted cosine similarity, which is defined as a normalized inner product between neuronal responses right after the first training and those after training on new patterns (see Methods in Supplementary Material). The adjusted cosine similarity declined monotonically over time (Fig. 4C), implying that training on new patterns gradually degrades the learned patterns, as in other online learning algorithms.

However, not every pair of digits shows a significant difference between their input signals and representations in the middle layer in lower order PC projects (see the digit-to-digit comparisons in Fig. S8 of Supplementary Material). To check whether the competition-based learning rule improves the classification performance for every digit, we considered a network model without a hidden layer, i.e. input units connect directly to output units with least-square-fitted connections. This input-direct least-square fitting resulted in 82.4% correct classification, compared with the correct rate of 91.5% for a network model with a 200-unit hidden layer trained by the synaptic competition rule. The digit-wise comparisons in Fig. 4D suggest that the competition-based learning rule boosts separations between different digit classes. Furthermore, we compared classification performance between random input weights and input weights trained by synaptic competition. We sampled the random weights from the standard normal distribution. Synaptic competition outperformed random weight sampling in all digit classes, although the overall correct rate was as high as 85.8% for the latter. These results prove the advantage of synaptic competition as an algorithm doing pattern separation.

## Discussion

### Intuitive explanation of the learning rule

Although both LTD and synaptic competition can separate noninterfering patterns, only the latter rule can split patterns with substantial overlaps. The existence of a synaptic connection indicates its importance for categorizing some learned patterns. When the network learns a new pattern, the critical difference between the two rules is while LTD removes connections from weakly responding presynaptic neurons, synaptic competition eliminates connections to weakly responding postsynaptic neurons (see Fig. 1B and C). Therefore, the latter learning rule organizes a layer of postsynaptic neurons specialized for learned or novel patterns. In other words, such connections are selectively removed that do not produce sufficient specialization of postsynaptic responses. If LTP cooperates with synaptic competition, the network is thought to balance strong responsiveness to similar input patterns and response sensitivity to individual differences between the patterns. (see Fig. 1I). This cooperation helps the neural network to differentiate input patterns without prior information about classes or labels.

Furthermore, the maturation rule described in Eq. 10 helps less-connected neurons to specialize for a new pattern. The maturation rule imposes an upper bound on the total strength of synaptic connections on a postsynaptic neuron. Hence, the rule is essential for suppressing the endless growth of synaptic connections on highly active postsynaptic neurons, which survive synaptic competition at high chances. Eq. 14 predicts that in the absence of maturation effects, those postsynaptic neurons already better connected will further dominate the competition for new connections. These neurons will prevent other neurons from being activated. Altogether, the competition rule enhances the specialization of neuronal responses, while the maturation rule induces a fair competition.

### Comparison with other models

The present work investigated how a feedforward network with synaptic competition for presynaptic resources separates interfering input patterns in an unsupervised way. In particular, our results suggest that synaptic competition greatly enhances pattern separation when the size of sample data is small. In addition, we exposed our network model to each sample of input patterns only once during training, aiming at mimicking biologically realistic situations of online learning. Although the biological details of the synaptic competition are oversimplified, we formulated the core effects of our weight-updating rule, namely, the maturation and competition terms, based on recent biological observations (9). Therefore, we believe that our model captures the key properties of synaptic competition in biological neuronal networks.

Although learning by competition was reported in the literature, the model of synaptic competition shown in this study is novel in several aspects. A recent study proposed an algorithm in which different input patterns compete for projections to hidden layer neurons (43). Although the algorithm worked well in numerical experiments with the MNIST data set, some input neurons developed projections to many hidden layer neurons, implying that many input patterns activate these input neurons. This overload to particular neurons makes such a model biologically less plausible. The model was also not tested on online learning with a small number of samples.

By contrast, the competition term in the present learning rule functions as a subtractive normalization term, suppressing the sum of outgoing weights of each input neuron. This prevents the overload of input neurons. Other models for synaptic competition mainly focused on competition for postsynaptic resources. For instance, in a model (e.g. (44)), restrictions of resources in postsynaptic neurons may limit the sum of input weights on each hidden layer neuron. However, while the model shed light on the dynamics of physiological processes, it did not clarify the crucial role of synaptic competition in pattern separation. In another model (45), homeostatic depression (HD) was modeled by a learning rule competing for postsynaptic resources. The model suggests that LTP and HD jointly maximize the online learning capacity. However, the model did not deal with interfering patterns and pattern separation. The use of inhibitory circuits to compete for responses to input patterns gives another way to separate patterns by shifting the tuning curves of excitatory neurons (46). Their rule targets a neural mechanism of pattern separation different from the synaptic competition learning rule, as it requires prior knowledge of input patterns in constructing an appropriate competitive inhibitory feedback.

Some numerical studies on adult neurogenesis explored the computational functions of neuronal turnover. We previously reported a network model in which input weights on newborn DG neurons were assigned randomly after their births (38). However, the model ignored long-term plasticity, which is known to play a nonnegligible role in developing dendrites during the 4 to 6 weeks of the birth of new neurons. Another model considered long-term plasticity by using a Hebbian learning rule (36). However, the learning rule did not explicitly take the role of synaptic competition into account, missing the important characteristics of adult neurogenesis. Yet another model involved the concept of synaptic competition (37), but did not reveal its nontrivial roles such as those studied here. Although our simplified model is far from a full spec model of neurogenesis, our numerical analyses revealed the essential contribution of synaptic competition to the discrimination of similar but different stimuli, a vital brain function. Furthermore, our model predicted that disabling synaptic competition impairs pattern separation only when stimuli are similar (see Fig. 2D to F and Fig. S3 of Supplementary Material). This prediction is testable by future experiments.

### Potential application in machine learning

Neural networks play a pivotal role in machine learning and artificial intelligence. Investigations of neural pathways in the brain have often inspired novel ideas and techniques in machine learning. For example, the neocognitron invented by Fukushima was inspired by the anatomical organization and neural responses of visual pathway (47). Since then, a rich family of neural networks, namely, the convolutional neural network, has been proposed for a variety of tasks, ranging from classification (48) to style transfer (49). Recently, the coding of a modern machine learning algorithm for natural language processing was found to be consistent with experimentally observed brain signals (50). These studies demonstrate how the discoveries and knowledge learned from the brain could advance methods in machine learning.

In the current study, neurons in the hidden layer compete for input signals, and this competition is activity-dependent. This means that synaptic competition processes input activity patterns based on the nature of the patterns. Thus, synaptic competition is an unsupervised algorithm for training input weights of hidden units without specifying particular purposes. Further, the comparison between models shown in Fig. 3C to E reveals that synaptic competition significantly improves classification performance when the sample data size is small. We may intuitively understand this remarkable feature of presynaptically driven synaptic competition through its basic property. When synapses compete for input patterns, they will be more successful if they can discriminate minor differences between different input patterns. We speculate that this pressure enables our network model to discriminate mutually interfering input patterns. This ability of synaptic competition is quite useful for online training, which requires simultaneous learning and data collection. Furthermore, in Fig. 3C to E, we obtained the results for synaptic competition just after one training cycle, whereas those for backpropagation required five cycles. These results suggest that the synaptic competition rule can generate efficient and effective coding of input patterns. Although the particular network architecture shown in Fig. 3A may not suit other tasks, the learning rule proposed in this study can be widely used.

In sum, we have demonstrated that synaptic competition can enhance pattern separation. It suppresses the effect of interference across similar input patterns. Our results suggest that synaptic competition makes similar patterns more distinguishable during the sensitive period of newly born neurons in adult neurogenesis. We have also shown that synaptic competition gives a promising algorithm for unsupervised learning in real-world classification tasks. Our findings will contribute not only to advancing the understanding of adult neurogenesis but also to developing novel online machine-learning methods for difficult classification tasks.

## Supplementary Material

pgad161_Supplementary_Data

## Data Availability

The source code underlying this article is available on GitHub at https://github.com/fccaa/Synaptic_Competition.

## Preprints

A preprint of this article is published at https://doi.org/10.1101/2022.09.15.508061.
