# Supplementary material for: Competition on presynaptic resources enhances the discrimination of interfering memories
Source: PNAS Nexus. 2023 May 15;2(6):pgad161. doi: 10.1093/pnasnexus/pgad161 (PMC10235910; doi:10.1093/pnasnexus/pgad161)
Supplement: pgad161_Supplementary_Data [file pgad161_supplementary_data.pdf]

# Supplementary Material for the Manuscript Entitled "Competition on Presynaptic Resources Enhances the Discrimination of Interfering Memories"

Chi Chung Alan Fung (alan.fung@cityu.edu.hk) and Tomoki Fukai (tomoki.fukai@oist.jp)

## Methods

### The Neuronal Network and Training Patterns Generated from Fundamental Patterns

The network is defined by

$$y_i = \mathrm{relu}\left(\sum_j w_{ij} x_j + \zeta_i\right), \tag{S1}$$

where $x_j$ is the input value at input node $j$, $y_i$ is the activation of node $i$, $w_{ij}$ is the coupling between them and $\zeta_i$ is the input noise.

The sample patterns were constructed from fundamental patterns:

$$\xi^{(i)} = \bar{\xi}^{(\varphi(i))} + \eta\delta\left(i\right), \tag{S2}$$

where $\bar{\xi}$s are fundamental patterns given in Eq. (1) or Eq. (8), $\varphi$ is the random mapping from $i$ to $\{0, 1, 2, 3\}$, $\eta = 0.1$ is the magnitude of the noise, and $\delta$ is a noise vector with entries between 0 and 1.

### Training Process with Different Training Rules

There are 1000 training samples were generated by Eq. (S2) for training processes aimed to show the difference between training rules with long-term potentiation, long-term potentiation with long-term depression, and long-term potentiation with the synaptic competition.

In training, all input weights were initially zero. In each iteration, training patterns were input into the network in a one-by-one manner. After each input, the neuronal activity $y$ will be updated. Then input weights were updated according to $\Delta w$ in the equations accordingly. In particular, input weights were trained in only one iteration.

The parameters used in various studies are presented in the following. For long-term synaptic potentiation, in Eq. (2), the learning rate, $\gamma$, was fixed to be 0.1. For long-term synaptic potentiation and long-term synaptic depression, in Eq. (6), the learning rate, $\gamma$, was also fixed to be 0.1, and the level of long-term depression, $\theta$, was chosen to be 0.25. For long-term synaptic potentiation with synaptic competition, in Eq. (13), the learning rate, $\gamma$, was fixed to be 0.1. In Eq. (11), the threshold for maturation, $\Theta$, was chosen to be 1.0.

### The Classification-task Comparison

#### The Data Set

The hand-written digit data set MNIST consists of 70000 digits [1]. The size of the input is $28 \times 28 = 784$ pixels. The numbers of training samples were 5000, 10000, and 50000. The rest of the digits were reserved to be in the testing set. The corresponding labels of those digits were one-hot coding. One-hot means that the expected output of the feedforward network is an array having 10 binary entries and only one of the entries is one.

### The Extended Data Set

We have also tested the learning rule on a data set combined of MNIST and Kuzushiji-MNIST [2]. There are 140,000 patterns from 20 classes. The input size is also $28 \times 28 = 784$ pixels. The number of training samples were 5000 and 50000. The corresponding labels of those input patters were one-hot coding.

### Neuronal Network Trained by Synaptic Competition

In the training of the input weights shown in Fig. 3A, the training digits were inputted into the network one by one. After each input, the input synaptic weights will be updated by the synaptic competition rule. The parameters for the synaptic competition rule were $\gamma = 0.01$ and $\Theta = 1.0$.

The output weights, $\mathbf{w}^{\text{out}}$, is determined by least-square fitting. Let $\mathbf{x}^{\text{training}}$ is a matrix whose columns are training input patterns, and $\mathbf{w}$ is the matrix for the trained input weights. We have

$$\mathbf{y}^{\text{training}} = \left[\mathbf{w}\mathbf{x}^{\text{training}}\right]_+ , \tag{S3}$$

where $[\cdot]_+$ is an element-wise rectified linear function. By using the neuronal activity $\mathbf{y}^{\text{training}}$ and the expected one-hot coding $\mathbf{z}^{\text{training}}$, we can determine the output weights $\mathbf{w}^{\text{out}}$ as follow.

$$\mathbf{w}^{\text{out}} = \mathbf{z}^{\text{training}}\, \mathbf{y}^{\text{training}\,T} \left(\mathbf{y}^{\text{training}}\, \mathbf{y}^{\text{training}\,T}\right)^{-1} . \tag{S4}$$

In the assessment of classification performance, digit patterns from the testing pool were inputted into the input layer of the network. The coding of the output layer will be simply the winner-take-all rule. The correct rate was calculated by the ratio of the number of successful predictions and the size of the testing pool.

### Multi-layer Perceptrons Trained by Back-propagation

The multi-layer perceptron network used in this study was constructed by an interface, namely Keras [3]. The back-end behind Keras is Tensorflow. The middle layer of the network was constructed by a number of rectified linear units. The output layer is constructed by softmax functions.

In the training phase, the network was trained by the training routine included in Keras. The back-propagation was done by stochastic gradient descent (SGD) optimizer. The loss function was categorical cross-entropy. There were five training epochs on the whole training set before testing. During testing, the calculation was similar. The correct rate is calculated by the ratio between successful predictions and the size of the testing pool.

## Adjusted Cosine Similarity

To measure the degrade of neuronal representations of learned pattern due to new learning, we define adjusted cosine similarity for the purpose. Cosine similarity between two vectors (say $\vec{v}$ and $\vec{u}$) is defined by a normalized dot product:

$$\text{Cosine Similarity of } \vec{v} \text{ and } \vec{u} \equiv \frac{\langle \vec{v}, \vec{u} \rangle}{|\vec{v}|\,|\vec{u}|}. \tag{S5}$$

However, since the signal is non-negative, this measure is largely offsetted. To discount this effect, we used the "adjusted cosine similarity" for comparison. The adjusted cosine similarity is defined by

$$\begin{aligned} \text{Adjusted Cosine Similarity of } \vec{v} \text{ and } \vec{u} \equiv & (\text{Cosine Similarity of } \vec{v} \text{ and } \vec{u}) \\ & - (\text{Cosine Similarity of shuffled } \vec{v} \text{ and shuffled } \vec{u}) . \end{aligned} \tag{S6}$$

This measure can tell a fair comparison across difference digits.

## Supplymemtary Figures

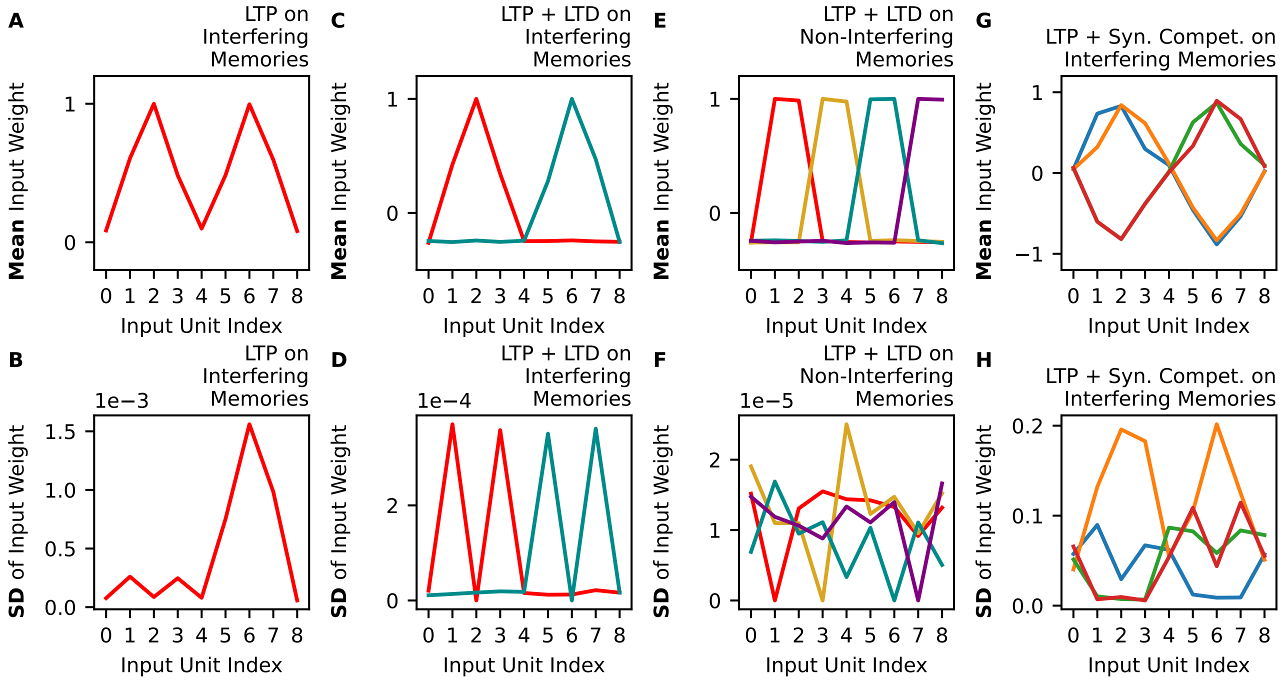


Figure S1: Means and standard deviations of input connection weight profiles trained by different long-term potentiation (LTP) and long-term potentiation with long-term depression (LTP + LTD) under different scenarios. **A**. & **B**.: Mean and standard deviation (SD) of input weight profiles trained by long-term potentiation on overlapping patterns. **C**. & **D**.: Mean and standard deviation (SD) of input weight profiles trained by long-term potentiation with long-term depression on overlapping patterns. **E**. & **F**.: Mean and standard deviation (SD) of input weight profiles trained by long-term potentiation with long-term depression on distant patterns. **G**. & **H**.: Mean and standard deviation (SD) of input weight profiles trained by long-term potentiation with synaptic competition on overlapping patterns.

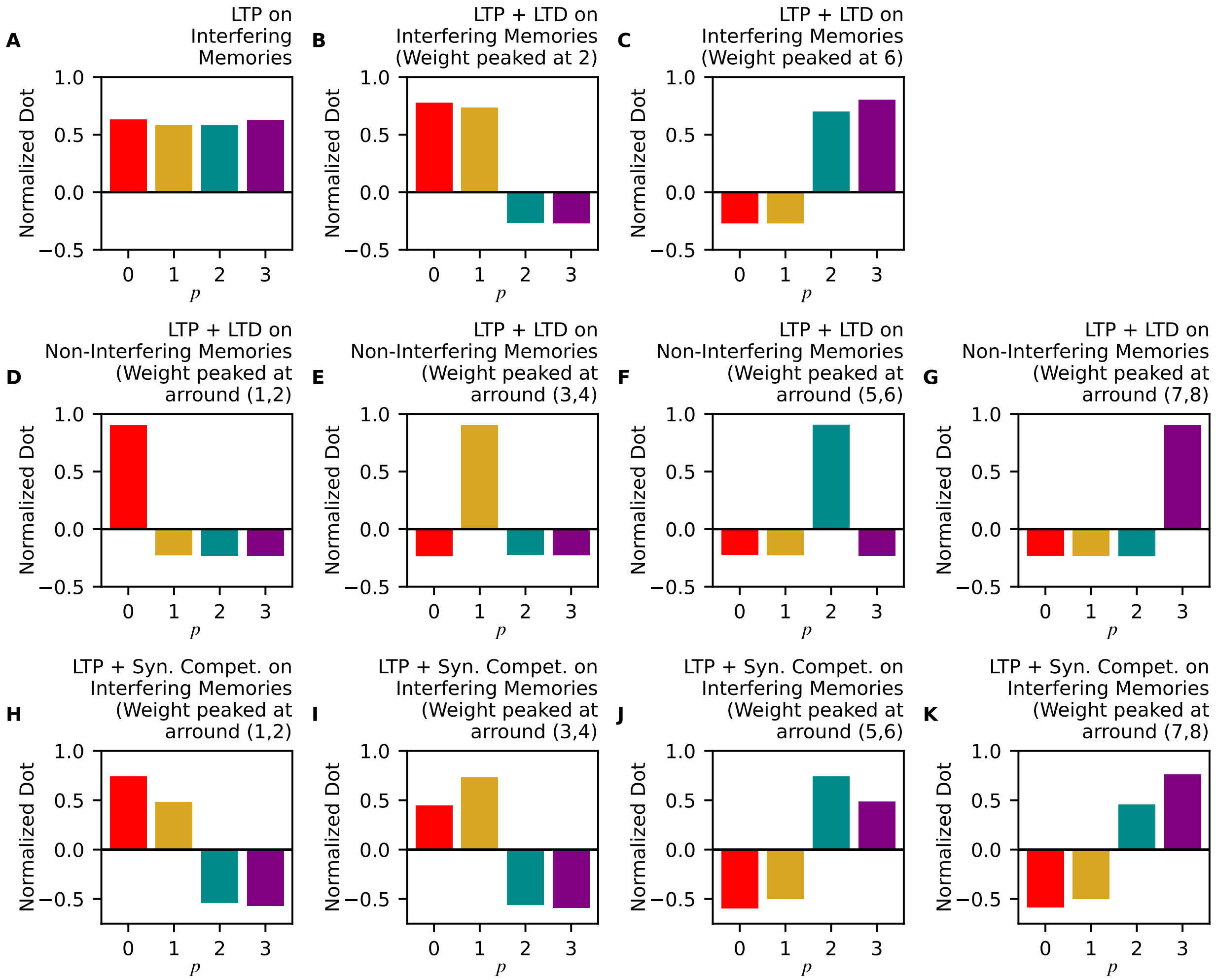


Figure S2: Similarity between fundamental patterns for training and post-training input weights. **A**. The normalized dot products between fundamental patterns ($p = 0, 1, 2, 3$) and input weights after training by the LTP rule on the interfering training data set (see Fig. 1F). **B-C**. The normalized dot products between fundamental patterns ($p = 0, 1, 2, 3$) and input weights after training by the LTP and LTD on the interfering training data set (see Fig. 1G). **D-G**. The normalized dot products between fundamental patterns ($p = 0, 1, 2, 3$) and input weights after training by the LTP and LTD on the non-interfering training data set (see Fig. 1H). **H-K**. The normalized dot products between fundamental patterns ($p = 0, 1, 2, 3$) and input weights after training by the LTP and synaptic competition on the interfering training data set (see Fig. 1I).

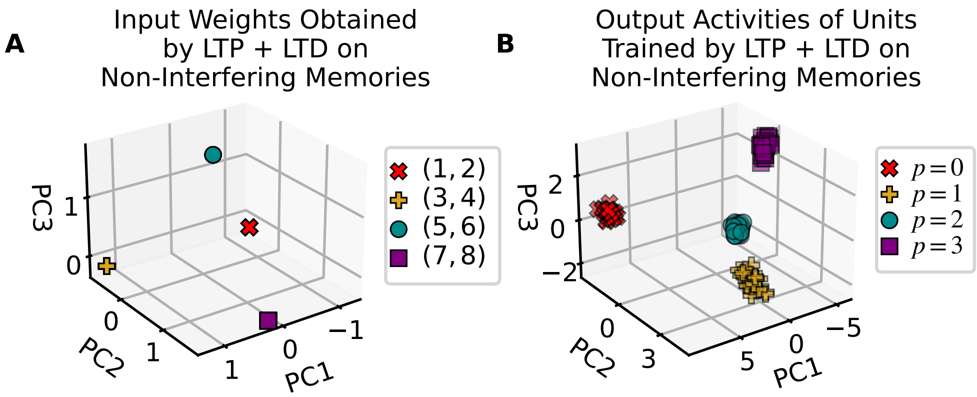


Figure S3: Principal component analysis (PCA) of input synaptic weights and activities of output units after training on the non-interfering patterns. **A**. The input weights trained by LTP and LTD are projected on the first three leading principal components (PCs), i.e., PC1, PC2, and PC3. **B**. The outputs of neurons encoding different preferred fundamental patterns are plotted in the 3D space of the leading PCs.

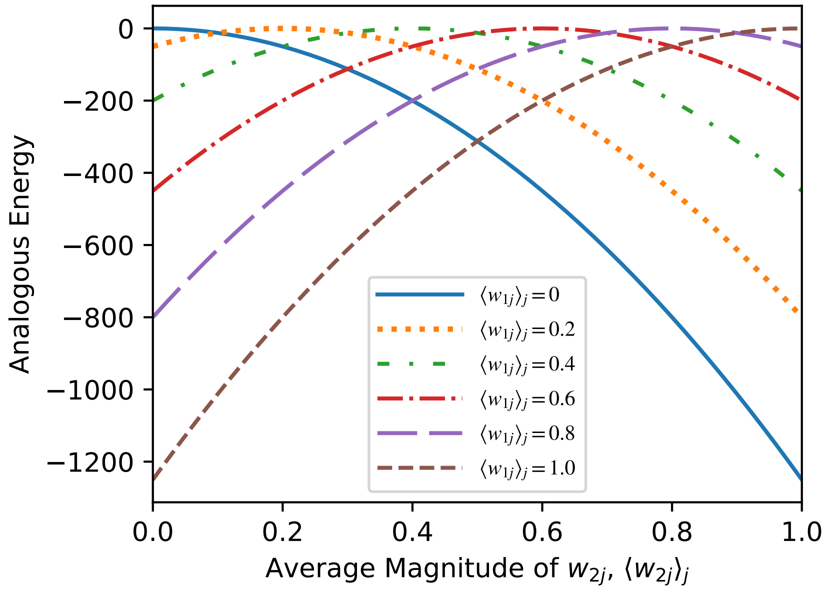


Figure S4: Local energy functions of the average synaptic weights for $i = 2$ under the influence of neuron $i = 1$.

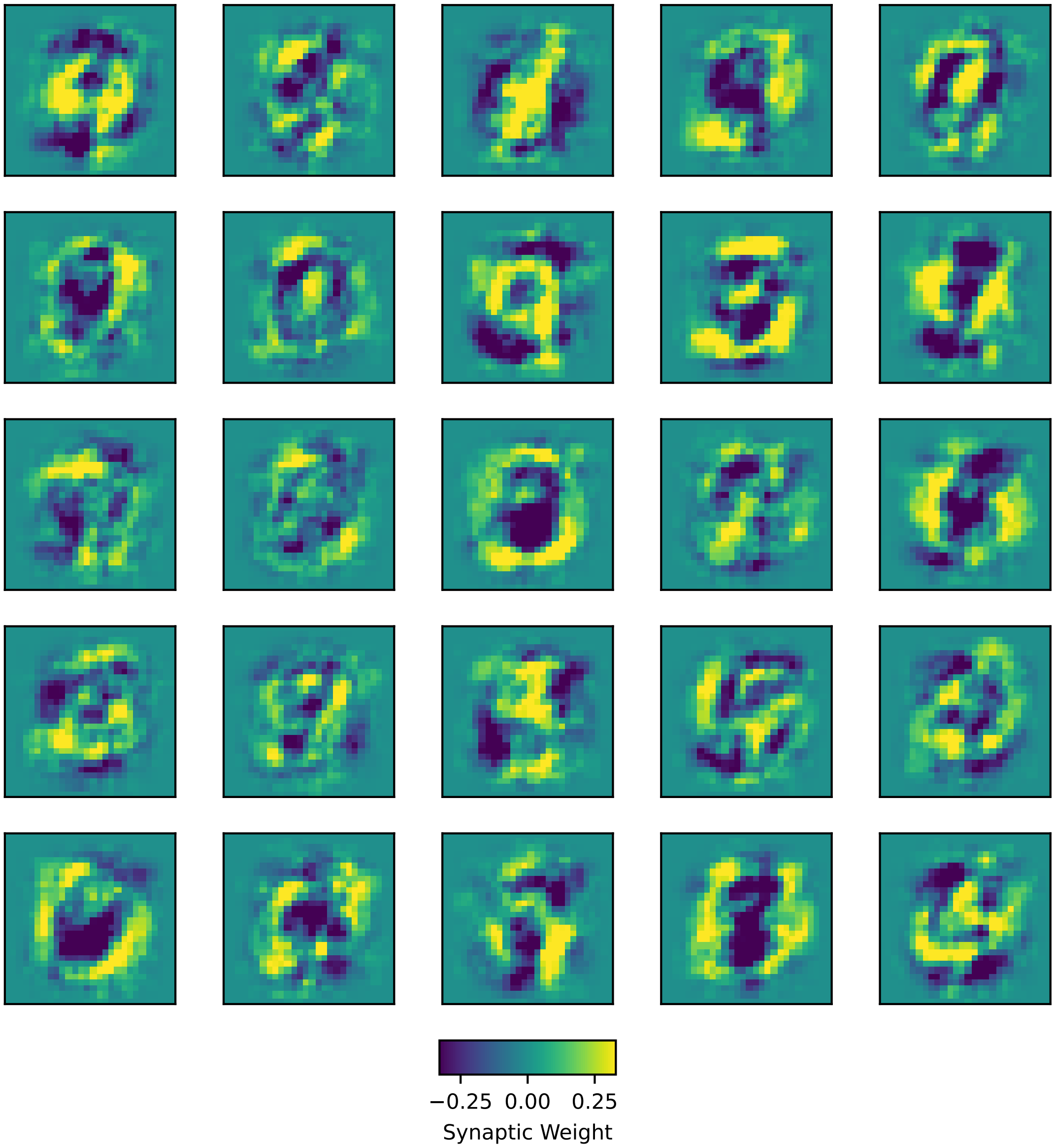


Figure S5: Additional examples of trained input weights on the middle layer of the competition-based network model in Fig. 3A.

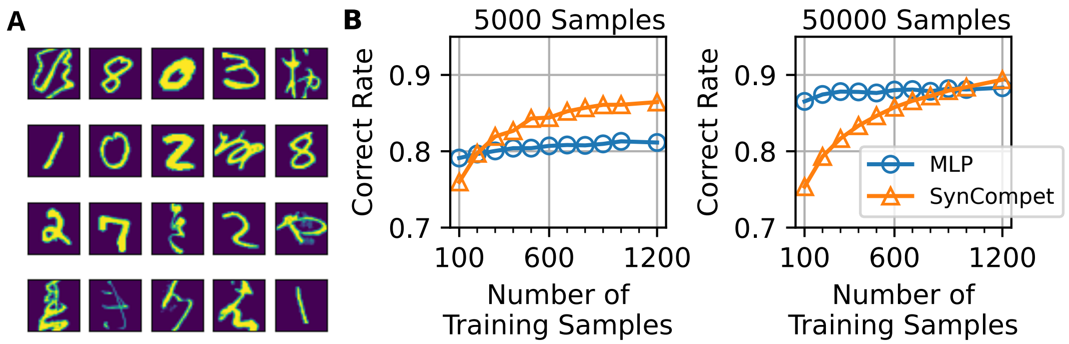


Figure S6: **A**. Example training patterns from the data set combined of MNSIT and Kuzushiji-MNIST. **B**. Correct rates of the competition-based model and MLP. The two networks were trained with 5000 (Left), and 50000 (Right) samples from the combined dataset.

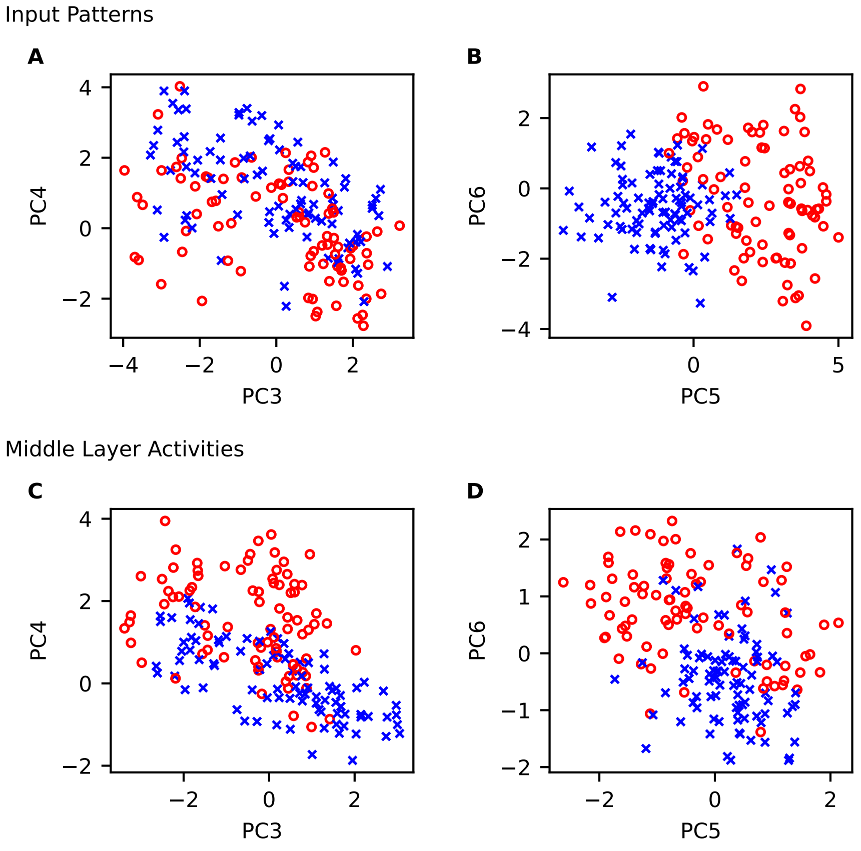


Figure S7: **A**. & **B**.: Higher principal-component (PC) projections of input patterns of hand-written digits 0 and 8. **C**. & **D**.: Higher principal-component (PC) projections of neuronal activities of a neuronal network corresponding to hand-written digits 0 and 8. The neuronal network was trained by synaptic competition.

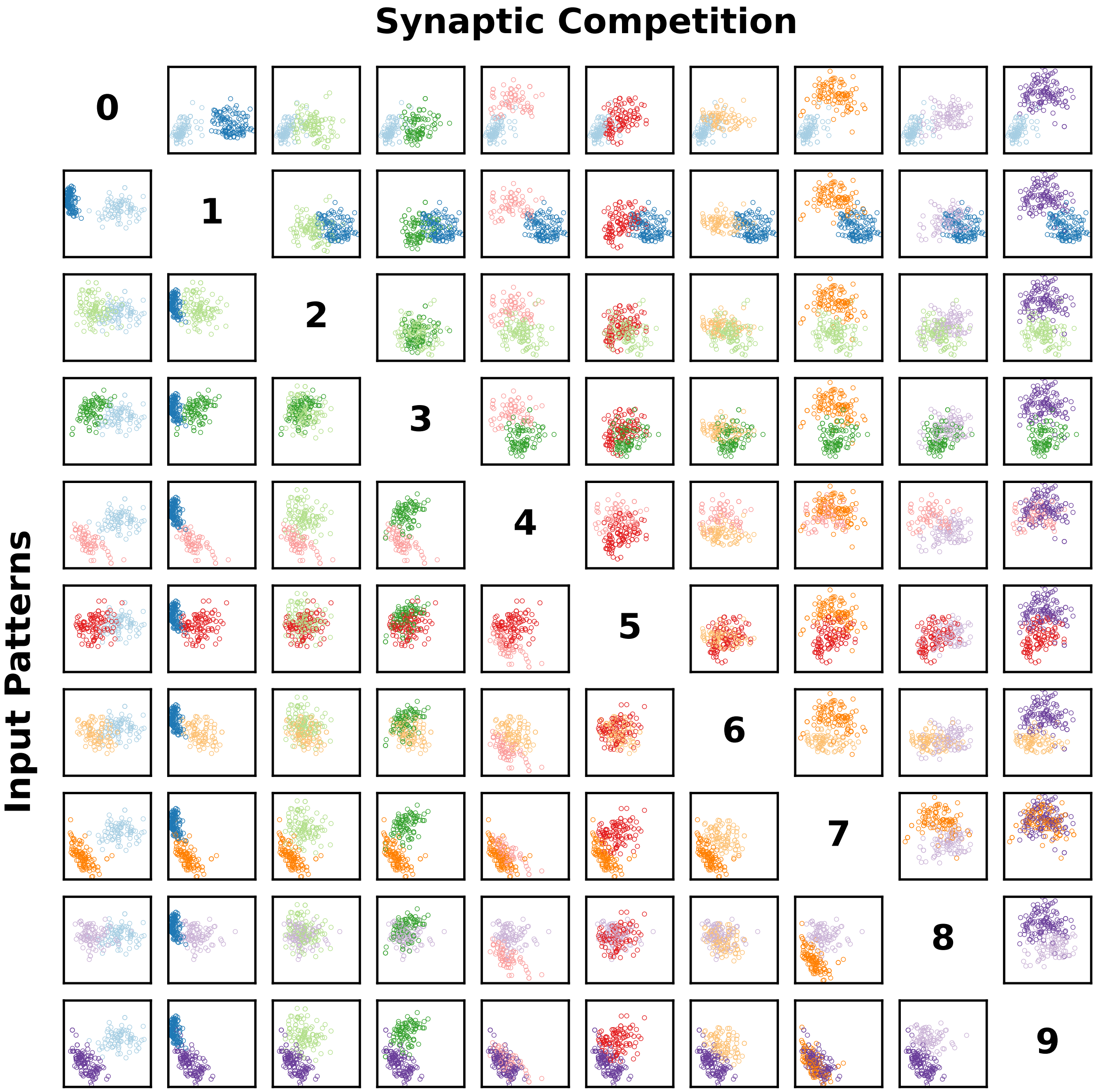


Figure S8: Comparisons of first and second principal components across digits. Lower triangle: principal components of input patterns. Upper triangle: principal components for activities of hidden units trained by synaptic competition. Horizontal axis: first principal components. Vertical axis: second principal component.

## References

[1] Yann LeCun, Corinna Cortes, and CJ Burges. Mnist handwritten digit database. *ATT Labs [Online]. Available: http://yann.lecun.com/exdb/mnist*, 2, 2010.

[2] Tarin Clanuwat, Mikel Bober-Irizar, Asanobu Kitamoto, Alex Lamb, Kazuaki Yamamoto, and David Ha. Deep learning for classical japanese literature, 2018.

[3] Francois Chollet et al. Keras. https://github.com/fchollet/keras, 2015.
